# The influence of acute lifestyle changes on NAFLD evolution in a multicentre cohort: a matter of body composition

**DOI:** 10.1038/s41387-024-00294-2

**Published:** 2024-05-27

**Authors:** Marcello Dallio, Moris Sangineto, Mario Romeo, Marina Cipullo, Annachiara Coppola, Simone Mammone, Giuseppe Di Gioia, Mario Masarone, Marcello Persico, Gaetano Serviddio, Alessandro Federico

**Affiliations:** 1 Hepatogastroenterology Division, Department of Precision Medicine, University of Campania “Luigi Vanvitelli”, Naples, Italy; 2 University Center for Research and Treatment of Liver Diseases (C.U.R.E.), Liver Unit, University of Foggia, Foggia, Italy; 3 Department of Medicine and Surgery, “Scuola Medica Salernitana”, Internal Medicine and Hepatology Unit, University of Salerno, Salerno, Italy

**Keywords:** Obesity, Preclinical research

## Abstract

**Background:**

Unhealthy lifestyles represent a key element fueling Non-alcoholic fatty liver disease (NAFLD) onset and worsening. We aimed to evaluate the effects of forced acute lifestyle changes on NAFLD evolution.

**Methods:**

187 NAFLD patients were followed two years pre- and two years during the lockdown social restrictions in three Italian medical centers. For each patient, biochemical, clinical, non-invasive liver fibrosis, nutritional, and body composition data were collected.

**Results:**

An increase in fats and carbohydrate intake associated with impaired weekly physical activity during the lockdown was demonstrated as well as an increase in body mass index and waist-hip-ratio (*p* < 0.0001 for all). Total cholesterol, low-density lipoprotein, high-density lipoprotein, triglycerides, glucose, insulin, homeostatic model assessment for insulin resistance, and transaminases worsened during the lockdown (glucose: *p* = 0.0007; *p* < 0.0001 for the others). Moreover, NAFLD fibrosis score, liver stiffness, and controlled attenuation parameter were also impaired during the same period (*p* < 0.0001 for all). The bioelectrical impedance analysis (BIA) evidenced an increase of fat mass (FM), and a reduction of free fat mass (FFM) and body cell mass (BCM) (*p* < 0.0001 for all). The lockdown overall hepatocellular carcinoma (HCC) and Milan-out HCC occurrence revealed Hazard Ratio (HR): 2.398, 95% Confidence Interval (CI):1.16–5, *p* = 0.02, and HR:5.931, CI:2–17.6, *p* = 0.008 respectively. A liver disease stage and comorbidities independent association between both the assessed outcomes and body composition analysis in terms of mean values and variation (T1–T2 Δ) was demonstrated.

**Conclusions:**

The acute lifestyle changes impacted NAFLD evolution via body composition modifications negatively influencing the HCC occurrence.

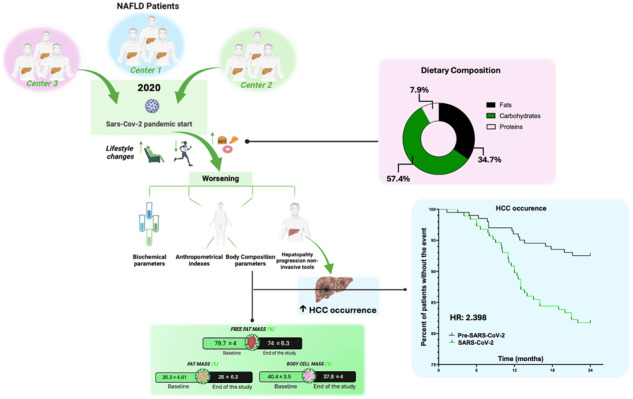

## Introduction

Non-alcoholic fatty liver disease (NAFLD) embraces a spectrum of pathological conditions ranging from non-alcoholic steatosis (NAFL) and steatohepatitis (NASH) to liver fibrosis and cirrhosis, rapidly becoming the most common cause of chronic liver damage and hepatocellular carcinoma (HCC) worldwide [1]. The projections reveal an alarming scenario, predicting an exponential increase in incidence and prevalence [2] with a very hard socioeconomic burden, considering the current unavailability of a defined pharmacological treatment [3–5].

NAFLD frequently onsets in the Metabolic Syndrome (MS) context, a condition in which the disruption of the glycolipid metabolism, synergically with low-grade systemic inflammation, promotes the development of related comorbidities such as type 2 diabetes mellitus (T2DM), obesity, arterial hypertension, and dyslipidemia [4, 6].

A huge number of scientific findings suggest an entangled correlation between incorrect lifestyle behavior and chronic dysmetabolic MS-related disorders [7, 8], including NAFLD [9, 10]. Relevantly, in a large cross-sectional study, prolonged sitting time was associated with increasing prevalence of NAFLD, independently from decreased physical activity level [11], as well as long desk working hours were significantly associated with an increased incidence of disease in a cohort of lean individuals [12], pointing out the crucial role of sedentary life in promote, on its own, NAFLD genesis and progression. More generally, the adoption of sedentary habits, simultaneously with dietary regimens based on high-caloric nutrients, by configuring a dramatic pathogenetic scenario where insulin resistance (IR) plays a predominant role, promotes NAFLD onset and worsening [13]. In contrast, as largely supported by various clinical studies, the combination of physical exercise with caloric restriction (“dual method”), determines great benefits in terms of NAFLD prevention, by improving systemic IR, increasing energy expenditure, and reducing lipid overload [14, 15]. In this sense, healthy habits appear to be crucial for the maintenance of balanced body composition which in turn prevents the NAFLD onset, by increasing Free Fat Mass (FFM) and reducing Fat Mass (FM) [16]. Furthermore, healthy lifestyles and weight loss were proven to be effective also in controlling liver fibrosis development and progression, which currently remains the most important prognostic factor in this context [17].

Considering this and the lack of specific approved drugs for NAFLD, the changes in diet and lifestyle represent the exclusive (non-drug based) treatment strategies, as well as an essential benchmark to design a tailored therapeutic approach, as decennially recommended by the clinical practice guidelines (CPG) [18–20]. However, in the last decade, the cumulating evidence on the efficacy of “dual method”-based NAFLD management and the adoption of these measures on a large scale have allegorized trains traveling at two different speeds on parallel tracks [21]. Several economic interests fueling the current “consumeristic” society, also configuring an environment where different stimuli negatively impact NAFLD patients’ awareness of the disease, thus reducing their therapeutic compliance, have represented, and currently, constitute relevant obstacles to the successful implementation of lifestyle-changes-based measures in clinical practice [21–23]. Therefore, although valid therapeutic weapons are currently available, the “war” to face the global spread of NAFLD remains an open social health question.

In the last weeks of 2019, the severe acute respiratory syndrome coronavirus 2 (SARS-CoV-2), caused the outbreak of a new world epidemic beginning in Wuhan, the capital of Hubei province, China and rapidly spreading globally [24]. Italy was one of the first Western countries seriously affected by this pandemic in early 2020. To counteract the virus in the national context, the government imposed a severe lockdown from 11 March 2020, obliging the immediate stop of commercial activities, schools and universities, restaurants, and outdoor and indoor people meetings [25]. For most of the Italian population, it was the first experience of a strict lockdown due to health reasons and determined the change in various daily personal attitudes [25]. The lifestyle modifications included, among others, the combination of reduced physical activity and unhealthy eating habits which realized a detrimental mixture contributing to a positive energy balance whose main metabolic consequences in terms of IR-worsening and total body fat increase have been widely reported [26]. However, even if the long-term perpetuation of unhealthy lifestyle behaviors is well recognized to hurt the NAFLD natural history, the consequences of their rapid onset have been only partially explored [27]. Therefore, we aimed to evaluate the effects of the confinement and related lifestyle changes on NAFLD evolution.

## Materials and methods

### Experimental design

We performed a four-year retrospective study on a NAFLD cohort from January 2018 to January 2022, dividing the study period by the beginning of the lockdown social restrictions in January 2020 [Baseline (T0); intermediate (end of the pre-pandemic period–January 2020) (T1); end of the study (January 2022) (T2)]. We routinely followed up the enrolled patients with clinical, biochemical, and imaging assessments in accordance with the current CPG and presented the data as mean values of the recordings that occurred during the specific period of observation.

For the entire length of the study, we screened and eventually recorded HCC occurrence by using ultrasonography assessments following CPG [28].

**Fig. 1:**
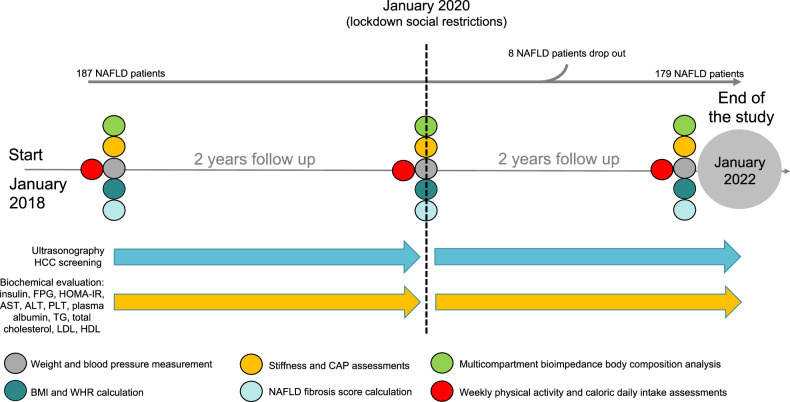
Study design flowchart. ALT Alanine Aminotransferase, AST Aspartate aminotransferase, BMI Body Mass Index, CAP Controlled Attenuation Parameter, FPG Fasting Plasma Glucose, HCC Hepatocellular carcinoma, HDL High-density lipoprotein, HOMA-IR Homeostatic model assessment for insulin resistance, LDL Low-density lipoprotein, NAFLD Non-alcoholic fatty liver disease, PLT Platelet count, TG triglycerides, WHR Waist-hip ratio.

The study’s experimental design is reported in Fig. 1.

The study’s primary endpoint was to assess the impact of the SARS-CoV-2 spread-related lifestyle changes on body composition analysis and metabolic syndrome components worsening.

The secondary endpoint was to assess the impact of the pandemic on HCC occurrence, as well as, shed light on the pandemic risk factors for HCC onset.

The entire study protocol was registered on the NIH U.S. National Library of Medicine database of clinical trials (NCT05416970). The “STrengthening the Reporting of Observational Studies in Epidemiology” (“STROBE”) checklist is reported in Supplementary File 1.

### Patients

This retrospective multicenter longitudinal study is in compliance with the ethical guidelines of the Declaration of Helsinki (1975) and was approved by the ethical committee of the University of Campania “L. Vanvitelli” in Naples (prot n. 15.04-20220010000, 29^th^ Mar 2022).

Between January 2018 and January 2022, patients affected by NAFLD based on clinical, biochemical, imaging, and histology, in accordance with CPG diagnostic criteria, and continuously followed by three centers (Hepato-gastroenterology Division of the University of Campania “Luigi Vanvitelli, Internal Medicine and Hepatology Unit of the University of Salerno and Institute of Internal Medicine of the University of Foggia), were enrolled in the present study, after signing informed consent.

The inclusion criteria were age between 18 and 80 years and NAFLD diagnosis.

Exclusion criteria were the presence of chronic inflammatory diseases such as inflammatory bowel disease, acute or chronic kidney disease, rheumatoid arthritis, systemic lupus erythematosus, or other major systemic diseases or tumors, ongoing infections, alcohol or drug abuse history, other etiologies of chronic liver damage, previous HCC diagnosis, use of hepatoprotective drugs, the administration of insulin (i.e., T2DM enrolled affected patients were all insulin-naïve), and psychological/psychiatric problems that could have invalidated the informed consent.

Medical history was collected, and alcohol consumption by using the complete Alcohol Use Disorders Identification Test (AUDIT-C) questionnaire was longitudinally assessed; medications (in particular, therapies for the dysmetabolic comorbidities: T2DM, obesity, arterial hypertension, and dyslipidemia) were opportunely classified and longitudinally recorded, and drug abuse was investigated.

Blood pressure (BP) [systolic (SBP) (mmHg) and diastolic (DBP) (mmHg)] were directly measured by an expert physician according to the American Heart Association (AHA) recommendations [29]. In detail, using an aneroid sphygmomanometer (GIMA, 32725, London) with an adequate cuff size (for arm circumference of 27 to 34 cm, the “adult” size of 16 × 30 cm was considered) and stethoscope (GIMA, 32719), the auscultatory method based on the Korotkoff technique, in which peculiar sound modifications describe 5 different phases, was adopted to collect SBP and DBP. To implement the predictive power of measurements, for everyone, multiple blood pressure determinations were obtained by the same physician: two readings were taken at intervals of at least 1 min, and the average of those readings was used to represent the patient’s BP; if there was >5 mm Hg difference between the first and second readings, additional (1 or 2) readings were obtained, and then the average of these multiple readings was used [29]. Weight (kg) and height (m) were also determined, and the Body Mass Index (BMI) was calculated by dividing the weight (kg) by the square of height (m) as Quetelet’s index (kg/m^2^) [30].

Waist (cm) and hip circumference (cm) were directly measured by using a tape measure (Xrten, B08DTG7RQ8), and the waist-to-hip ratio (WHR) was calculated. Waist circumference was recorded at the minimum circumference between the iliac crest and the rib cage, whereas hip circumference was recorded at the maximum circumference over the buttocks [30]. For each patient, these measurements were obtained in duplicate with subjects standing dressed in underwear, and both were rounded to the nearest 0.5 cm [30].

The following biochemical variables were recorded during the entire study period: insulin, fasting plasma glucose (FPG), the homeostatic model assessment for IR (HOMA-IR), aspartate aminotransferase (AST), alanine aminotransferase (ALT), platelet count (PLT) and plasma albumin (PA), total cholesterol, triglycerides (TG), low-density lipoprotein (LDL), high-density lipoprotein (HDL). Insulin levels (μU/mL) were measured enzymatically using commercially available kits (R&D Systems, Minneapolis, MN), whereas, AST (U/L), ALT (U/L), FPG (mmol/L), LDL and HDL, and TG using a colorimetric assay kit (AST, ALT: Amplite 13801/13803; FPG: Thermo Fisher Scientific EIAGLUC; LDL and HDL: Sigma Aldrich MAK045; TG: Sigma Aldrich MAK266). PA (g/dL) was assessed by using the BCA protein assay (Sigma Aldrich QPBCA), as a validated protein quantification method. PLT count (x10^3^/μL) was performed with an automated hematology analyzer by using a suspension of blood cells passing through a small orifice along with an electric current of the Beckman Coulter analyzer [C11137 - DxI 9000 Analyzer, Beckman Coulter, Inc^©^].

HOMA-IR was calculated using the following formula: fasting insulin (μU/mL) × FPG (mmol/L)/22.5 [31].

### Food intake, alcohol assumption, and physical exercise assessments

At the end of the pre-pandemic period, as well as at the end of the study, the food intake relative to a complete week, including working days and the weekend, was recorded, and evaluated by using the software WinFood, Medimatica s.r.l., Martinsicuro, Italy. Based on the quantity and quality of foods consumed, the program estimates the percentage of macronutrients and micronutrients in each food and elaborates the daily energy intake in terms of Kcal per day referring to the caloric amount as specifically related to carbohydrates, fats, and proteins dietary proportions. The complete elaboration of intakes shows the list of diet components, the ratio among components, the calories, and the subdivision into breakfast, lunch, and dinner. The availability of a list of diet components, for each patient, allowed an expert dietician to compute the dietary composition, in terms of fat types (saturated, monounsaturated, and polyunsaturated) and carbohydrate types (monosaccharides, disaccharides, and polysaccharides) proportion.

Alcohol assumption was evaluated with a standardized pre-codified questionnaire (AUDIT-C test): the quantity of daily alcohol intake was calculated based on a “drink” that corresponds to about 12 g of pure ethanol [32].

Physical exercise was assessed by a specific medical-assisted questionnaire (Supplementary File 2).

### Non-invasive liver disease progression status evaluation: liver stiffness measurement, controlled attenuation parameter assessment, and calculation of NAFLD fibrosis score

Liver stiffness measurement (LSM) was obtained by transient elastography (TE), which was performed by using the FibroScan ® version 502 (Echosens, Paris, France) with M and XL probes [33]. We decided to use the XL probe when the ultrasonography measured distance between the skin to liver capsule resulted in greater than 2.5 cm and/or when BMI was >30. FibroScan ® was performed by an expert physician obtaining 10 acceptable measurements (defined as successful LSM), with the maximum number of attempts set at 20. The criteria proposed by Boursier et al. were used to consider the measurement “very reliable” (IQR/M ≤ 0.1), “reliable” [0.1 < IQR/M ≤ 0.3 or IQR/M > 0.3 with LS median < 7.1 kilopascal (kPa)], or “poorly reliable” (IQR/M > 0.3 with LS median ≥ 7.1 kPa) [33].

The controlled attenuation parameter (CAP) measures ultrasonic attenuation in the liver at 3.5 MHz using signals acquired by the FibroScan® M and XL probes based on physical principles described elsewhere [34]. The CAP was measured only on validated measurements according to the same criteria used for LSM [33, 34].

NAFLD fibrosis score (NFS) was determined by using the formula: -1.675 + 0.037 × age (years) + 0.094 × BMI (kg/m^2^) + 1.13 × T2DM (yes = 1, no = 0) + 0.99 × AST/ALT ratio−0.013 × PLT (×10^9^/L)−0.66 × PA (g/dL) [35].

### Multicompartment bioimpedance body composition analysis assessment

A multifrequency bioelectrical impedance analysis (BIA) system (InBody, Seoul, Korea) was used to perform the body composition assessment. For the analysis, two electrodes on the right foot and hand were placed. The patients assumed the supine position and were relaxed for at least 15 minutes before the assessment that was performed in duplicate.

The mean values of the two different measurements were used for the analysis. For this purpose, a gentle voltage waveform at 50 kHz was passed through the body of each enrolled patient from the hand electrodes to the foot ones. Using the reactance (Xc), resistance (R), and phase angle [arctangent (Xc/R) × (180/π)] the BIA system, thanks to a series of types of machinery algorithms elaborated the total body water (TBW), the intracellular and extracellular body water (ICW/ECW), the FFM, the FM, body cell mass (BCM) expressed both in percentage and kilograms (Kg). Skeletal Muscle Mass Index (SMMI) was calculated by dividing the Skeletal Muscle Mass (SMM) by the square of the height (m^2^).

### Recording of HCC occurrence and definition of HCC overall and Milan-out criteria

The screening for HCC was adequately performed by using ultrasonography assessments, as well as HCC diagnosis was opportunely achieved respecting the European Association for Study of the Liver (EASL) CPG: contrast-enhanced ultrasound (CEUS) showing HCC imaging hallmarks (LiRADS 5) defined this neoplasm [28]. On the diagnosis, to stage HCC occurrence, Milan criteria were adopted [36]: patients presenting 1 lesion ≥ 2 cm and ≤ 5 cm or up to 3 lesions, each ≥1 cm and ≤ 3 cm, without evidence of vascular invasion or extra-hepatic metastases, were defined as “HCC Milan-in criteria”, contrariwise individuals not respecting the abovementioned criteria were considered “HCC Milan-out criteria”. Hence, “HCC overall” represented the sum of “HCC Milan-in criteria” and “HCC Milan-out criteria”.

### Statistical analysis

Baseline demographics were summarized using descriptive statistics. Continuous data were described as mean and standard deviations, while categorical variables were summarized as n (%).

The Kolmogorov-Smirnov test for normality was performed to evaluate if parametric or non-parametric analysis should be applied. Wilcoxon signed ranks test and t-test for dependent groups were performed to compare continuous variables between two times of observation.

The Kruskal-Wallis test or ANOVA test with post-hoc Tukey analysis, in the case of non-normal or normal distribution respectively, were performed to compare the continuous variables among three times of observation.

Time-to-event analyses on HCC occurrence and Milano-out staging at the diagnosis were performed by using the Kaplan-Meier method and the Log-rank test for the curve comparison considering a *p*-value < 0.05 as statistically significant. The proportional hazard assumption was verified using the Schoenfeld residuals test.

The odds ratios (OR) of the study variables on the just mentioned events were calculated considering the confounding variables (age, sex, BMI, T2DM, SARS-CoV-2 infection, and LSM) by using logistic regression models. Sensitivity analyses were performed stratifying the overall sample by age, sex, BMI, type 2 diabetes, SarsCoV2, and LSM.

Statistical significance was defined as p < 0.05 in a two-tailed test with a 95% confidence interval. Statistical Program for Social Sciences (SPSS®) vs.18.0 was used to perform the analysis.

## Results

### Baseline characteristics of the study cohort

A cohort of 187 NAFLD patients was enrolled and followed up for four years. One-hundred seventy-nine of 187 patients were followed for the entire study evaluation; eight patients dropped out during the pandemic period due to HCC-related deaths.

The study cohort was composed of 97 females (51.9%) and 90 males (48.1%) with a baseline average age of 46.1 years. Seventy-four (39.6%) patients were affected by T2DM and 62 (33.1%) contracted SARS-CoV-2 infection during the observation period. One-hundred-four enrolled patients (55.6%) were affected by LSM < 10 kPa NAFLD (F0-2 according to Metavir) while 83 (44.4%) LSM ≥ 10 kPa, of whose 53 (28.3%) were classified as compensated advanced chronic liver disease (cACLD) based on LSM > 15 kPa [37].

The clinical characteristics, as well as anthropometric indexes, biochemical parameters, and non-invasive tools for fibrosis/steatosis (NFS, LSM, and CAP) of the study population, are reported in Table 1.

**Table 1:** Clinical features of the study population and the assessment of anthropometric indexes, biochemical parameters, and non-invasive tools among the three-time points evaluations.

Clinical characteristics of the study population							
Sex (frequency distribution)				SARS-CoV-2 infection during the observation period (frequency distribution)			
Male (n and %)		Female (n and %)		Yes (n and %)		No (n and %)	
90 (48.1%)		97 (51.9%)		62 (33.1%)		125 (66.9%)	
**Dysmetabolic comorbidities and relative medications administration** (frequency distribution)							
**Type 2 Diabetes Mellitus (T2DM)**				**Medications administration in T2DM affected patients (n:74)**			
Yes (n)	No (n)	Yes (%)	No (%)	No (n and %)	Yes (n and %)	**Class of medication**	Patients using the drug (n and %)
74	113	39.6%	60.4%	3 (4.1%)	71 (95.9%)	**Metformin**	32 (45.1%)
						**Sulfonylureas**	20 (28.2%)
						**Gliptins**	19 (26.7%)
**Dyslipidemia**				**Medications administration in dyslipidemia affected patients (n:83)**			
Yes (n)	No (n)	Yes (%)	No (%)	No (n and %)	Yes (n and %)	**Class of medication**	Patients using the drug (n and %)
83	104	44.4%	55.6%	9 (10.9%)	74 (89.1%)	**Statins**	43 (58.1%)
						**Ezetimibe**	22 (29.7%)
						**Statin/Ezetimibe**	9 (12.1%)
						**Fibrates**	15 (20.3%)
**Obesity**				**Medications administration in obesity affected patients (n:98)**			
Yes (n)	No (n)	Yes (%)	No (%)	No (n and %)	Yes (n and %)	**Class of medication**	Patients using the drug (n and %)
98	89	52.4%	47.59%	82 (83.7%)	16 (16.3%)	**DPP4- I**	9 (56.2%)
						**GLP-1 agonist**	5 (31.3%)
						**Intestinal lipase-I (orlistat)**	2 (12.5%)
**Essential arterial hypertension**				**Medications administration in hypertension affected patients (n:92)**			
Yes (n)	No (n)	Yes (%)	No (%)	No (n and %)	Yes (n and %)	**Class of medication**	Patients using the drug (n and %)
92	95	49.2%	50.8%	17 (18.5%)	75 (81.5%)	**Diuretics**	37 (49.3%)
						**ACE-I**	22 (29.3%)
						**CCB**	11 (14.6%)
						**Beta-Blockers**	13 (17.3%)
						**Sartans**	19 (25.3%)

Anthropometric indexes, biochemical parameters, and non-invasive tools							
Variables (mean ± SD)		Baseline (T0: January 2018)	Intermediate (T1: January 2020)	End of the study (T2: January 2022)	Comparison between the three-time points evaluations		
					Time-points	95% CI	*p*-value
Height (m)		1.69 ± 0.07	/	/	/	/	/
Weight (kg)		79.6 ± 9.6	79.4 ± 10.12	88.3 ± 14.9	T0 vs T1	0.4678 to 0.7754	0.82
					T0 vs T2	−8.966 to −4.42	**<0.0001**
					T1 vs T2	−9.035 to −4.659	**<0.0001**
SBP (mmHg)		132.1 ± 12	131.2 ± 9.4	130.5 ± 9.4	T0 vs T1	−0.7622 to 2.431	0.43
					T0 vs T2	−0.1017 to 3.314	0.07
					T1 vs T2	−0.7127 to 2.256	0.44
DBP (mmHg)		78.7 ± 10.9	78.4 ± 9.5	78.6 ± 8.4	T0 vs T1	−1.363 to 2.01	0.91
					T0 vs T2	−1.669 to 1.924	0.98
					T1 vs T2	−1.763 to 1.369	0.95
Body Mass Index		27.8 ± 2.2	27.7 ± 2.4	30.1 ± 4.6	T0 vs T1	0.1591 to 0.2752	0.8
					T0 vs T2	−3.05 to −1.453	**<0.0001**
					T1 vs T2	−3.083 to −1.541	**<0.0001**
WHR		0.97 ± 0.13	0.97 ± 0.13	1.13 ± 0.22	T0 vs T1	−0.020 to 0.022	0.99
					T0 vs T2	−0.201 to −0.120	**<0.0001**
					T1 vs T2	−0.201 to −0.122	**<0.0001**
WHR/BMI		0.035 ± 0.005	0.035 ± 0.004	0.039 ± 0.008	T0 vs T1	−0.001 to 0.001	0.774
					T0 vs T2	−0.004 to −0.002	**<0.001**
					T1 vs T2	−0.004 to −0.002	**<0.001**
Total cholesterol (mg/dL)		185.2 ± 17.2	183.8 ± 16.3	223.2 ± 28.2	T0 vs T1	−1.398 to 2.464	0.79
					T0 vs T2	−44.60 to −32.59	**<0.0001**
					T1 vs T2	−44.94 to −33.31	**<0.0001**
LDL (mg/dL)		118.9 ± 21.2	118.3 ± 20.05	162.5 ± 30.3	T0 vs T1	−3.011 to 4.105	0.929
					T0 vs T2	−49.55 to −37.7	**<0.0001**
					T1 vs T2	−49.88 to −38.47	**<0.0001**
HDL (mg/dL)		43.7 ± 11.8	43.7 ± 10.4	30.4 ± 9.2	T0 vs T1	−1.893 to 1.853	0.99
					T0 vs T2	11.04 to 15.66	**<0.0001**
					T1 vs T2	11.21 to 15.53	**<0.0001**
Triglycerides (mg/dL)		110.8 ± 26.2	111.2 ± 26.8	153.4 ± 43.8	T0 vs T1	−4.414 to 4.344	0.99
					T0 vs T2	−47.76 to −33.7	**<0.0001**
					T1 vs T2	−47.47 to −33.95	**<0.0001**
Insulin (μU/mL)		22.01 ± 11.4	23.02 ± 11.8	24.4 ± 12.6	T0 vs T1	−1.602 to 1.310	0.8143
					T0 vs T2	10.76 to 14.70	**0.0003**
					T1 vs T2	10.63 to 14.39	**0.0003**
Glucose (mmol/L)		4.8 ± 1.6	4.9 ± 1.7	5.1 ± 1.8	T0 vs T1	−1.402 to 1.420	0.8366
					T0 vs T2	10.26 to 12.80	**0.0007**
					T1 vs T2	10.42 to 12.19	**0.0007**
HOMA-IR		5.13 ± 3.85	5.92 ± 3.91	6.15 ± 4.41	T0 vs T1	−1.301 to 1.520	0.7892
					T0 vs T2	11.76 to 12.70	**<0.0001**
					T1 vs T2	10.13 to 13.59	**<0.0001**
AST (U/L)		40.9 ± 15.1	40.1 ± 13.5	54.5 ± 15.6	T0 vs T1	−1.494 to 3.044	0.699
					T0 vs T2	−16.65 to −10.56	**<0.0001**
					T1 vs T2	−17.33 to −11.43	**<0.0001**
ALT (U/L)		70.5 ± 15.3	69.4 ± 15.7	87.4 ± 21.9	T0 vs T1	−1.406 to 3.672	0.1354
					T0 vs T2	−20.32 to −13.56	**<0.0001**
					T1 vs T2	−21.94 to −14.21	**<0.0001**
PLT (x10^3^/μL)		157.7 ± 34.5	158.2 ± 34.4	153.1 ± 40.8	T0 vs T1	−2.108 to 1.092	0.7320
					T0 vs T2	6.491 to 15.09	0.0552
					T1 vs T2	7.488 to 15.11	0.1334
Albumin (g/dL)		3.6 ± 0.3	3.6 ± 0.3	3.5 ± 0.5	T0 vs T1	−0.008 to 0.035	0.3064
					T0 vs T2	0.104 to 0.207	0.1225
					T1 vs T2	0.093 to 0.191	0.0576
NFS		−0.732 ± 0.946	−0.633 ± 0.991	−0.034 ± 1.134	T0 vs T1	−0.205 to 0.007	0.072
					T0 vs T2	−0.828 to −0.566	**<0.0001**
					T1 vs T2	−0.743 to −0.453	**<0.0001**
LSM (kPa)		9.85 ± 4.5	9.82 ± 4.38	11.68 ± 5.21	T0 vs T1	−0.073 to 0.117	0.847
					T0 vs T2	−2.162 to −1.499	**<0.0001**
					T1 vs T2	−2.197 to −1.508	**<0.0001**
CAP (dB/m)		290.8 ± 32.8	291.0 ± 33.6	334.3 ± 45.7	T0 vs T1	−1.788 to 1.799	>0.99
					T0 vs T2	−50.16 to −36.7	**<0.0001**
					T1 vs T2	−50.07 to −36.78	**<0.0001**

*ACE-I* Angiotensin-converting enzyme inhibitors, *ALT* alanine aminotransferase, *AST* aspartate aminotransferase, *CCB* Calcium channel blockers, *DPP4-I* dipeptidyl peptidase inhibitors, *GLP-1* glucagon-like peptide 1, *DBP* Diastolic Blood Pressure, *CAP* controlled attenuation parameter, *dL* deciliter, *HDL* high-density lipoprotein, *HOMA-IR* homeostasis model assessment for insulin resistance, *kPa* Kilopascal, *L* liter, *LSM* Liver stiffness measurement, LDL Low-density lipoprotein, M meter, *mmol* millimoles, *mmHg* millimeters: millimeter of mercury, *NFS* NAFLD Fibrosis Score, *PLT* platelets, *SBP* Systolic Blood Pressure, *SD* standard deviation, *U* unit, *WHR* waist-hip ratio, *μ* micro, *n* number. The Kruskal-Wallis test or ANOVA test with post-hoc Tukey analysis, in the case of non-normal or normal distribution respectively, were performed to compare the continuous variables among three observation times. Statistically significant differences (*p* < 0.05) among the three periods are reported in bold.

### Nutritional and dietary habits modifications

The changes in dietary habits assessed during the study time points were reported in Supplementary Table 1 and as a percentage of carbohydrates (with relative types: monosaccharides, disaccharides, and polysaccharides), fats (with relative types: saturated, monounsaturated, polyunsaturated) and proteins dietary proportions in Supplementary File 3.

The time spent in physical activity was significantly decreased at T2 in comparison to the T1 and T0 evaluations (*p* < 0.0001 both). Contrariwise, considering the same time points of assessment, a significant increase in the daily caloric intake was reported from the T0 and T1 evaluation to the T2 (*p* < 0.0001 both).

Simultaneously to such quantitative modifications, the macronutrients (carbohydrates, lipids, and proteins) also revealed significant changes in the dietary composition in terms of kcal/day: carbohydrates and lipids proportions significantly increased contrary to the significant decrease of proteins, comparing the T0 and T1 assessments with the end of the study (*p* < 0.0001 for all). Contrariwise, no statistically significant modification of the carbohydrate (monosaccharides, disaccharides, and polysaccharides) and fat types (saturated, monounsaturated, polyunsaturated) proportion, as well as AUDIT-C-assessed daily alcohol intake, was recorded for the entire length of the study.

### Modifications of the anthropometrical indexes, biochemical parameters, and hepatopathy progression non-invasive tools

A significant increase in BMI, WHR, and WHR/BMI comparing the T0 and T1 assessments with the end of the study (*p* < 0.0001 for BMI and WHR; *p* < 0.001 for WHR/BMI), whereas no significant changes in SBP and DBP were observed (Table 1).

Concerning the biochemical parameters, a significant increase of total cholesterol, LDL, TG, insulin, glucose, HOMA-IR, AST, and ALT in contrast with a decrease in HDL were highlighted (*p* < 0.0001 for all except for glucose: *p* = 0.005) (Table 1).

**Fig. 2:**
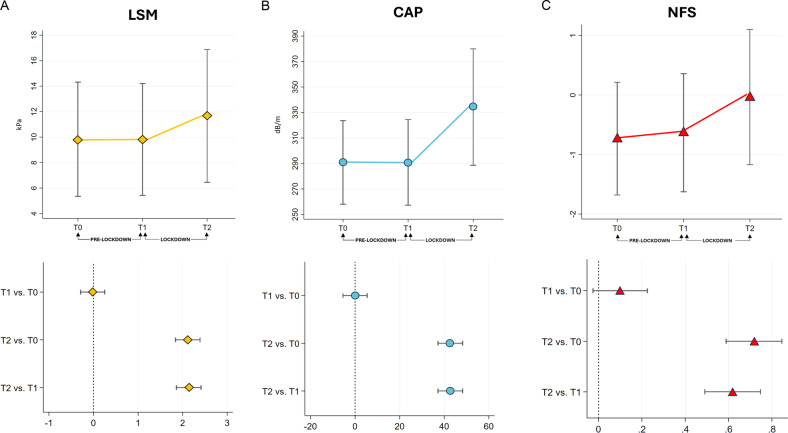
Hepatopathy progression non-invasive tools modifications. **A** LSM: Liver stiffness measurement, **B** CAP: Controlled attenuation parameter, **C** NFS: NAFLD Fibrosis score comparison among the three study time points by using ANOVA and Tukey post hoc analysis. kPa: Kilopascal; db/m: decibel/meter. *p < 0.0001.

Finally, the non-invasive tools for liver disease progression status revealed a worsening of NFS, LSM, and CAP comparing the T0 and T1 assessments with the end of the study (*p* < 0.0001 for all) (Table 1 and Fig. 2A–C).

### Modifications of body composition

A statistically significant increase of FM, both in Kg and percentage relative to the other body compartments, in contrast with a reduction of FFM percentage comparing the end of the study evaluation to the previous study time points was observed (*p* < 0.0001 for all). Consistently, by focusing the attention on the other body sub-compartments, a simultaneous significant reduction of extracellular mass (ECM) and BCM, both in Kg and percentage values, comparing the end of the study evaluation to the previous study time points, was observed (*p* < 0.001 for all) (Supplementary Table 2).

### HCC overall and HCC Milan-out criteria occurrence

**Fig. 3:**
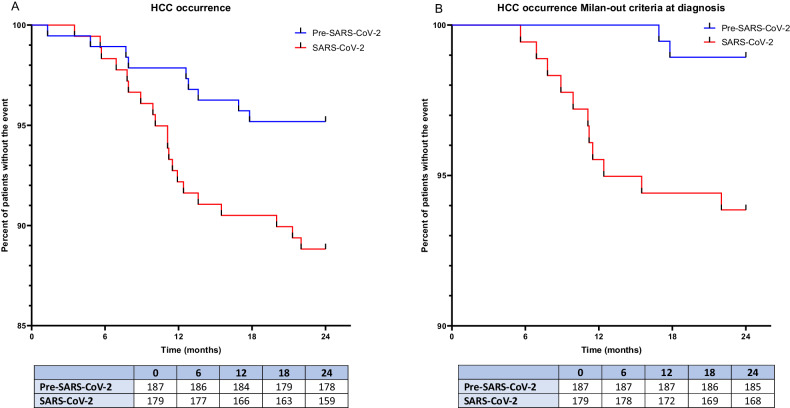
Hepatocellular Carcinoma occurrence in the period preceding the lockdown and during the lockdown. The Kaplan-Meier Log-Rank Test analysis comparing the HCC overall (**A**) and Milan-out criteria at diagnosis (**B**) occurrence pre- vs during the lockdown.

Notably, both the HCC overall and HCC Milan-out criteria occurrence significantly increased during the lockdown. From January 2018 to January 2020, we recorded HCC overall occurrence in 9 of 187 patients (HCC incidence rate: 4.8%) of which 2 developed HCC staged Milan-out criteria at diagnosis (HCC Milan-out incidence rate on the total of HCC occurrence: 22.2%). Relevantly, from January 2020 to January 2022, 20 of 179 patients developed HCC (HCC incidence rate: 11.1%), and among these, 11 presented HCC staged Milan-out criteria at diagnosis (incidence rate on the total of HCC occurrence: 55%). Due to the lockdown restrictions, 39 of 179 patients procrastinated the abdominal ultrasound in the context of HCC screening with a variable delay ranging from 30 to 112 days. However, among this subset of patients, no one developed HCC for the entire length of the study evaluation. In line with the above results, the Kaplan-Meier Log-Rank Test analysis on HCC overall occurrence and HCC staged Milan-out criteria at diagnosis occurrence during the lockdown revealed HR:2.39, 95% CI:1.16-5, *p* = 0.02, and HR:5.9, 95% CI:2-17.6, *p* = 0.008 respectively (Fig. 3A, B) (Supplementary Table 3A, B). Supplementary Table 4 reports the logistic regression analysis for both HCC overall and HCC staged Milan-out criteria at diagnosis concerning the lockdown period. As noticed, among the assessed parameters, contrarywise to biochemical ones apart from ALT (*p* = 0.045, OR: 1.03, 95% CI: 1.001-1.061) (insulin: *p* = 0.766, glucose: *p* = 0.85, HOMA-IR: *p* = 0.918, AST: *p* = 0.77, total cholesterol: *p* = 0.6, TG: *p* = 0.474, HDL: *p* = 0.665, LDL: *p* = 0.626), a significant association between various body composition indexes and HCC overall occurrence was highlighted. In particular, the FFM (both in Kg and percentage), the BCM (both in Kg and percentage), and the SMMI showed a negative association (*p* < 0.0001 for all, except for SMMI: *p* = 0.004), whereas the FM (both in Kg and percentage) was positively associated with the HCC overall occurrence (*p* < 0.0001 both) (Supplementary Table 4). Of notice, the LSM was negatively associated with the risk of HCC overall occurrence (*p* = 0.01), in contrast to the result that emerged in the pre-pandemic period (OR: 1.78, CI 95% 1.17-2.74, p < 0.007) during which, by the way, none of the abovementioned body composition parameters showed a statistically significant association with the HCC overall occurrence (HCC overall occurrence pre-lockdown-related lifestyles changes, FFM in Kg: *p* = 0.127, FFM percentage: *p* = 0.167, SMMI: *p* = 0.578, BCM in Kg: *p* = 0.324, BCM percentage: *p* = 0.549, FM in kilograms: *p* = 0.408, FM percentage: *p* = 0.168).

Regarding the HCC staged Milan-out criteria at diagnosis occurrence during the lockdown-related lifestyles changes period, contrarywise to biochemical ones (insulin: *p* = 0.08, glucose: *p* = 0.152, HOMA-IR: *p* = 0.091, AST: *p* = 0.071, ALT: *p* = 0.198, total cholesterol: *p* = 0.673, TG: *p* = 0.537, HDL: *p* = 0.669, LDL: *p* = 0.672), the analysis revealed a significant positive association with FM (both in Kg and percentage) (*p* = 0.002 both), and a negative one with the FFM (both in Kg and percentage), the BCM (both in Kg and percentage), and the SMMI (*p* = 0.01, *p* = 0.002, *p* = 0.01, *p* = 0.003, *p* = 0.04 respectively). The LSM emerged as negatively associated with the development of this outcome (*p* = 0.03) (Supplementary Table 4). None of the abovementioned biochemical, body composition, and non-invasive liver stiffness tools influenced the HCC staged Milan-out criteria at diagnosis occurrence during the pre-lockdown period.

**Fig. 4:**
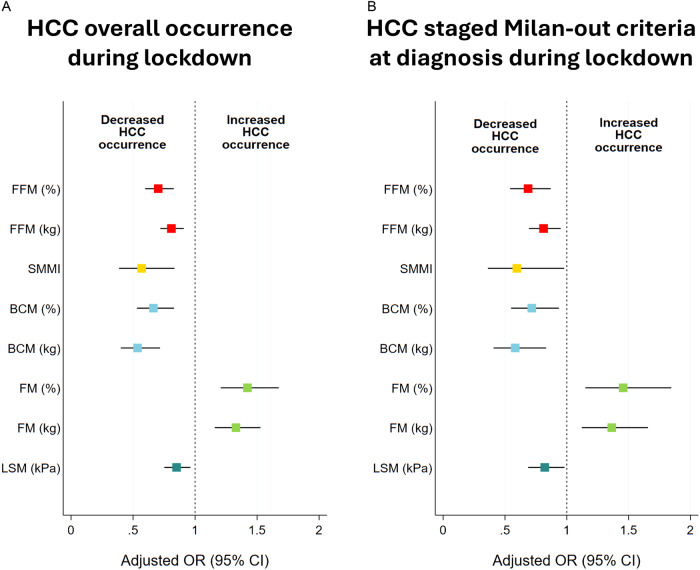
Body composition variables and relative association with Hepatocellular Carcinoma occurrence during the lockdown. Adjusted Odds Ratios for bioimpedance analysis factors’ variations on hepatocellular carcinoma (HCC) overall (**A**) and Milan-out criteria at diagnosis (**B**) occurrence during the lockdown period.

Finally, we investigated the association of the variation (T1 vs T2-Δ) for the aforementioned significant parameters with the risk of HCC overall and HCC staged Milan-out criteria at diagnosis occurrence during the lockdown period (Fig. 4 and Table 2). Consistently, Δ FM (Kg and percentage) was positively associated with both outcomes, whereas Δ FFM (Kg and percentage), Δ BCM (Kg and percentage), Δ SMMI, as well as Δ LSM, showed a negative association.

**Table 2:** Multinomial logistic regression analysis of the delta values (January 2020 vs end of the study assessments) of the parameters significantly associated with HCC overall and HCC staged Milan-out criteria at diagnosis occurrence during the lockdown.

Outcome: HCC overall occurrence during the lockdown			
Variable	Odds ratio	Confidence Interval (95%)	*p*-value
Δ FFM (%)	0.576	0.442-0.75	<0.0001
Δ FFM (kg)	0.782	0.693-0.882	<0.0001
Δ SMMI	0.570	0.351-0.926	0.02
Δ BCM (Kg)	0.457	0.326-0.639	<0.0001
Δ BCM (%)	0.564	0.437-0.729	<0.0001
Δ FM (Kg)	1.512	1.237-1.848	<0.0001
Δ FM (%)	1.737	1.334-2.262	<0.0001
Δ LSM (kPa)	0.398	0.248-0.638	0.0001
**Outcome: HCC staged Milan-out criteria at diagnosis during the lockdown**			
Δ FFM (%)	0.614	0.458-0.822	0.001
Δ FFM (kg)	0.796	0.578-0.862	0.001
Δ SMMI	0.456	0.225-0.923	0.02
Δ BCM (Kg)	0.207	0.066-0.645	0.007
Δ BCM (%)	0.483	0.326-0.713	0.0002
Δ FM (Kg)	1.417	1.127-1.781	0.003
Δ FM (%)	1.63	1.217-2.183	0.001
Δ LSM (kPa)	0.296	0.139-0.63	0.002

The odds ratios (OR) of the study variables on the just mentioned events were calculated considering the confounding variables (age, sex, BMI, T2DM, SARS-CoV-2 infection, and LSM).

Δ= variation January 2020 vs end of the study assessments, *BCM* Body cellular mass, *FFM* Free fat mass, *FM* Fat mass, *LSM* Liver stiffness measurement, *kPa* Kilopascal, *Kg* kilograms, *SMM* Skeletal muscle mass, *SMMI* Skeletal muscle mass index.

Interestingly, the delta values referred to the variations of the parameters comparing T0 vs T1 (Δ_2_) time points didn’t show a significant association with the risk of HCC occurrence during the pre-lockdown period (Δ_2_ FM in Kg: *p* = 0.426, Δ_2_ FM percentage: *p* = 0.248, Δ_2_ FFM in Kg: *p* = 0.186, Δ_2_ FFM percentage: *p* = 0.248, Δ_2_ BCM in Kg: *p* = 0.062; Δ_2_ BCM percentage: *p* = 0.195; Δ_2_ SMMI: *p* = 0.054; Δ_2_ LSM: *p* = 0.302].

The sensitive analysis for main outcomes stratified by age, sex, obesity status, diabetes status, and Sars-CoV-2 status is completely reported in Supplementary File 4.

## Discussion

In light of the latest etiologic revolution of chronic hepatopathies, the worldwide spread of metabolic-associated liver diseases and related comorbidities became one of the most important issues, that urgently needed to be addressed by the scientific community [38]. The lack of approved efficient medications, even more so considering the rapid evolution of the clinical picture up to HCC development, makes the NAFLD the current “hepatologist chimera”. During the last two years the population all over the world experienced a radical lifestyle change due to, from a medical point of view, the SARS-CoV-2 diffusion and the consequent governments forced rules to counteract the contagion impacting society [39, 40].

On this extraordinary background, the SARS-CoV-2 European spread represented the fuse able to trigger a series of unhealthy dietary and physical activity attitudes by which the NAFLD gains an inauspicious advantage. Our NAFLD cohort was followed up continuously for two years before and two years after the approval of lockdown social restriction rules, showing deep lifestyle changes in terms of total daily caloric intake and dietary composition as well as reduction of regularity and the time per week dedicated to physical exercise. As properly identified before in other contexts, the foreseeable effect of these duties was represented by a weight gain between 0.5 and 1.8 kg ( ± 2.8 Kg) after just 2 months of quarantine [25]. This happened particularly in specific population subsets experiencing already proven risk factors for this outcome, like increased snacking frequency, decreased water intake, emotional eating, decreased sleep quality, and being overweight/obese [41]. However, how this phenomenon impacts NAFLD, fueling its evolution, isn’t already properly known, due to the need to insert it in a more complex model of risk assessment that should take into consideration also the body composition analysis and the time frame for its modification. Taken together, our findings demonstrated the worsening of several metabolic parameters at the end of lockdown, in comparison to the previous periods (T2 vs T1 & T0). In particular, we pointed out the increase of the disease staging evaluated both via LSM and NFS calculation, also highlighting the increase of CAP values. Moreover, the association of this finding with the increase of several biochemical parameters, confirmed the derangement of the clinical picture during the lockdown, configuring a complex social-health scenario where liver disease worsening is promoted.

To our knowledge, our present study is the first observation of the influence of lifestyle behavior changes imposed by the lockdown on the NAFLD clinical progression and outcomes.

In a retrospective longitudinal study including 973 participants who underwent health check-ups between 2018 and 2020, Fujii et al. demonstrated the independent predictors for Metabolic dysfunction-associated fatty liver disease (MAFLD) development during the pandemic observation (2019–2020), resulting in the daily alcohol intake and, particularly for < 60 years old subjects, the proportion of participants who ate 2 times per day as statistically significant [27]. However, no data regarding the possible worsening of the disease in those patients presenting the diagnosis from the beginning of the observation were shown and in our setting the alcohol consumption assessment did not significantly change during the entire length of the study.

A mutual relationship between SARS-CoV-2 infection and NAFLD clinical picture has been previously demonstrated [42]. In this sense, the deleterious interplay of two inflammatory pathways, the one chronically active in NAFLD and the other acutely present during SARS-CoV-2 infection, could be considered the key pathogenetic mechanism of the liver damage observed in a subset of patients. Contemporarily, the underlying liver fibrosis might represent an additional and independent risk factor for severe SARS-CoV-2 illness, irrespective of metabolic comorbidities [43, 44]. Despite the SARS-CoV-2 infection in several enrolled patients during the pandemic spread, we would like to point out that it was not significantly associated with the impairment of the evaluated study parameters, nor with negative disease outcomes like HCC development or death.

No sequential data regarding the body composition analysis of NAFLD patients across the pandemic were published before. Here we showed a huge modification of body compartments of the enrolled population, particularly emphasizing the increase of FM (percentage and Kg) and a decrease of FFM (percentage) and BCM (percentage). Azoulay et al. in a recent observational study examined the change in body composition parameters of children and adolescents during the pandemic from May 15, 2020, until December 15, 2020. In this setting, they demonstrated in most of the enrolled subjects a relatively stable muscle-to-fat ratio (MFR) z-scores, increased in underweight (*p* = 0.05) and normal weight subjects (*p* = 0.008), but not in the overweight/obesity subgroup (*p* = 0.169). The multivariate linear regression identified socioeconomic position, pre-pandemic BMI z-scores, pre-pandemic MFR z-scores, and physical activity levels during the pandemic as predictors for delta MFR z-scores [45]. These findings shed light on the weakness, in terms of unhealthy body composition modifications, of that subset of the analyzed overweight population. Besides, the lack of dietary information and longer follow-up data in the specific NAFLD setting represent not at all negligible differences in comparison to our observation. In this sense, the qualitative and quantitative dietary information supporting our study coherently enhances the relevance of the obtained results. In terms of dietary composition, comparing the T0 and T1 assessments with the end of the study, carbohydrate and lipids proportions were significantly increased, contrariwise to the significant decrease of proteins. Previous research investigated the potential metabolically detrimental effects of simple carbohydrates (monosaccharides and disaccharides) on IR and hepatic steatosis, as well as suggested overeating saturated fats as a factor contributing to hepatic and visceral fat storage, in contrast with polyunsaturated fats intake promoting lean tissue in humans [46, 47]. Considering this evidence, the types of carbohydrate and fat proportions were evaluated in our study. Although, consistently, monosaccharides and disaccharides, as well as saturated fat proportions, appeared higher at T2 in comparison to the baseline, these variations were revealed not statistically significantly different, suggesting a more significant contribution of the “absolute” modifications of macronutrient class intake than a “relative” single one type to the evolving presented metabolic scenario.

Regarding physical exercise, in line with the previous evidence [45], a significant reduction of time spent in activity was revealed at T2 in comparison to the T1 and T0 evaluations. However, relevantly, various studies revealed sedentary habits as a detrimental factor for NAFLD, suggesting that prolonged sitting time is a crucial factor in promoting, on its own, NAFLD worsening and relative cardiometabolic comorbidities, independently from decreased physical activity level [11, 12]. Although validated questionnaires assessing simultaneously physical exercise and sedentary habits are currently available [48], to avoid *horror vacui* guiding the experimental approach, sitting time observation was not included and was not directly evaluated in our study design (NCT05416970). In this sense, considering the analyzed peculiar context where various social limitations imposed by pandemic-related government-established restrictions forced the study population to “home life”, consistently with the fact that home-sitting-practiced activities (including, among others, the use of social networks and internet gaming) [49–51] configured the totality of time spent on a “typical lock-down day”, the information regarding sedentary life can be easily deducible and approximable as the complementary of time spent in physical exercise, and thus not be considered missing in our research.

The prominent increase in HCC occurrence and the rate of “Milan-out” staging at the diagnosis during the lockdown in comparison to the previous study period were also highlighted.

As previously reported, the global incidence of NAFLD-related HCC before the pandemic ranged from 0.5% to 2.6% among patients with NASH cirrhosis and lower in non-cirrhotic NAFLD (approximately 0.1 to 1.3 per 1000 patient-years), making the showed findings alarming [52].

For this purpose, the impaired compliance to the screening program recommended by the CPG due to the risks from potential exposure and resource reallocation seemed to be not significant in influencing the observed findings [53]. On the contrary, the logistic regression model confirmed the LSM as an independent major risk factor for HCC occurrence in the pre-pandemic period, revealing the unforeseen role exerted by the body composition changes both on HCC occurrence and “Milan-out” staging at the diagnosis during the lockdown. Specifically, FM (Kg and percentage), FFM (Kg and percentage), SMMI, and BCM (Kg and percentage) were significantly associated with both the evaluated disease outcomes, independently from age, BMI, sex, T2DM, LSM, SARS-CoV-2 infection, and CAP.

Moreover, the OR values acquired even more relevance by the analysis of the delta modification (T2 vs T1) of the assessed parameters, reinforcing the association link between their worsening and the HCC. Unexpecting, the LSM during the pandemic resulted in less power in influencing the evaluated outcomes. The latter datum, however, must be carefully interpreted, because the greater part of newly diagnosed HCC during the pandemic developed in non-cACLD (n. 16/20) patients, and a prospective observational study on large cohorts could give further details.

Lastly, the chronic modification of the body composition represents an important prognostic determinant in the context of the HCC treatment in the case of systemic therapies administration like Lenvatinib or Sorafenib, as well as resective surgery and transcatheter arterial chemoembolization [54–57]. In light of the results, how and how much the rapid modifications of the body composition observed in our setting could impact NAFLD natural history, complications onset, and HCC evolution, also in terms of therapeutic response, remain an unmet open question.

The retrospective nature could represent the most important study limitation and a larger multicenter observational study could help to corroborate our results. A longer follow-up period could also improve the assessment of the real long-term impact of the pandemic in this context.

## Conclusions

The present study highlighted the negative impact of acute lifestyle changes on NAFLD evolution. During this relatively short lockdown period, a positive nutrition balance in terms of reduced physical exercise in association with an increased food intake mirrored the worsening of anthropometrical indexes, biochemical parameters, body composition compartments representation, liver fibrosis, and steatosis assessed by non-invasive tools.

An increased HCC (overall and “Milan-out” criteria at the diagnosis) occurrence during the lockdown was also observed. Relevantly, both outcomes were influenced by the body composition modifications without any impact of other variables, including impaired compliance. For this purpose, in light of the results, the body compartment analysis could represent a useful predictive tool for the NAFLD-tailored approach.

### Supplementary information

Supplementary

## Data Availability

The data that support the findings of this study are available from the corresponding author upon reasonable request.
