# Supplementary material for: The influence of acute lifestyle changes on NAFLD evolution in a multicentre cohort: a matter of body composition
Source: Nutr Diabetes. 2024 May 27;14:33. doi: 10.1038/s41387-024-00294-2 (PMC11130147; doi:10.1038/s41387-024-00294-2)
Supplement: Supplementary file 1 — Supplementary [file 41387_2024_294_MOESM1_ESM.pdf]

Supplementary File 1. STROBE Statement—checklist of items that should be included in reports of observational studies

	Item No.	Recommendation	Page No.	Comments
**Title and abstract**	1	(*a*) Indicate the study's design with a commonly used term in the title or the abstract	1	
		(*b*) Provide in the abstract an informative and balanced summary of what was done and what was found	2-3	
**Introduction**				
Background/rationale	2	Explain the scientific background and rationale for the investigation being reported	4	
Objectives	3	State specific objectives, including any prespecified hypotheses	4-5	
**Methods**				
Study design	4	Present key elements of study design early in the paper	5-6	
Setting	5	Describe the setting, locations, and relevant dates, including periods of recruitment, exposure, follow-up, and data collection	5-6	
Participants	6	(*a*) *Cohort study*—Give the eligibility criteria, and the sources and methods of selection of participants. Describe methods of follow-up<br>*Case-control study*—Give the eligibility criteria, and the sources and methods of case ascertainment and control selection. Give the rationale for the choice of cases and controls<br>*Cross-sectional study*—Give the eligibility criteria, and the sources and methods of selection of participants	6-7	
		(*b*) *Cohort study*—For matched studies, give matching criteria and number of exposed and unexposed<br>*Case-control study*—For matched studies, give matching criteria and the number of controls per case		
Variables	7	Clearly define all outcomes, exposures, predictors, potential confounders, and effect modifiers. Give diagnostic criteria, if applicable	7-8	
Data sources/ measurement	8*	For each variable of interest, give sources of data and details of methods of assessment (measurement). Describe comparability of assessment methods if there is more than one group	7-8	
Bias	9	Describe any efforts to address potential sources of bias	7-8	
Study size	10	Explain how the study size was arrived at	8-9	

Continued on next page

Quantitative variables	11	Explain how quantitative variables were handled in the analyses. If applicable, describe which groupings were chosen and why	9
Statistical methods	12	(*a*) Describe all statistical methods, including those used to control for confounding	9
		(*b*) Describe any methods used to examine subgroups and interactions	9
		(*c*) Explain how missing data were addressed	9
		(*d*) *Cohort study*—If applicable, explain how loss to follow-up was addressed<br>*Case-control study*—If applicable, explain how matching of cases and controls was addressed<br>*Cross-sectional study*—If applicable, describe analytical methods taking account of sampling strategy	9
		(*e*) Describe any sensitivity analyses	
**Results**			
Participants	13*	(a) Report numbers of individuals at each stage of study—eg numbers potentially eligible, examined for eligibility, confirmed eligible, included in the study, completing follow-up, and analysed	9-10
		(b) Give reasons for non-participation at each stage	9-10
		(c) Consider use of a flow diagram	N/A
Descriptive data	14*	(a) Give characteristics of study participants (eg demographic, clinical, social) and information on exposures and potential confounders	9-10 (+ Table 1)
		(b) Indicate number of participants with missing data for each variable of interest	9-10
		(c) *Cohort study*—Summarise follow-up time (eg, average and total amount)	9-10
Outcome data	15*	*Cohort study*—Report numbers of outcome events or summary measures over time	11-12-13
		Case-control study—Report numbers in each exposure category, or summary measures of exposure	
		Cross-sectional study—Report numbers of outcome events or summary measures	
Main results	16	(*a*) Give unadjusted estimates and, if applicable, confounder-adjusted estimates and their precision (eg, 95% confidence interval). Make clear which confounders were adjusted for and why they were included	9-10-11-12
		(*b*) Report category boundaries when continuous variables were categorized	9-10-11
		(*c*) If relevant, consider translating estimates of relative risk into absolute risk for a meaningful time period	9-10-11

Continued on next page

Other analyses	17	Report other analyses done—eg analyses of subgroups and interactions, and sensitivity analyses	N/A
**Discussion**			
Key results	18	Summarise key results with reference to study objectives	13-14-15-16
Limitations	19	Discuss limitations of the study, taking into account sources of potential bias or imprecision. Discuss both direction and magnitude of any potential bias	17
Interpretation	20	Give a cautious overall interpretation of results considering objectives, limitations, multiplicity of analyses, results from similar studies, and other relevant evidence	13-14-15-16-17
Generalisability	21	Discuss the generalisability (external validity) of the study results	15-16-17
**Other information**			
Funding	22	Give the source of funding and the role of the funders for the present study and, if applicable, for the original study on which the present article is based	N/A

*Give information separately for cases and controls in case-control studies and, if applicable, for exposed and unexposed groups in cohort and cross-sectional studies.

Note: An Explanation and Elaboration article discusses each checklist item and gives methodological background and published examples of transparent reporting. The STROBE checklist is best used in conjunction with this article (freely available on the Web sites of PLoS Medicine at http://www.plosmedicine.org/, Annals of Internal Medicine at http://www.annals.org/, and Epidemiology at http://www.epidem.com/). Information on the STROBE Initiative is available at www.strobe-statement.org.

Supplementary File 2. Food intake and Physical exercise assessment: explanation of the used tools.

Food Intake assessment

We recorded, by using a diet diary, the food intake for a complete week, including working days and weekends by using the software WinFood, Medimatica s.r.l.

Based on the quantities and qualities of food consumed, the program estimates the percentage of macronutrients and micronutrients in each food and elaborates the daily energy intake in terms of Kcal per day referring to the caloric amount as specifically related to carbohydrates, fats, and proteins dietary proportions.

Physical exercise assessment

Medical validated questionnaire and relative items assessing physical exercise practicing in enrolled patients.

**Questions/Items**	**Answer**	
Are you doing or have you ever done (in the last 2 years) sport in a continuative and regular way?	YES	NO
Have you changed your daily physical activity in the last 6 months?	NO	YES
If yes, has it enhanced or worsened?	Enhanced	Worsened
How many hours per week do you usually spend for physical exercise?	More than 150 minutes/week	Less than 150 minutes/week

Each patient was considered on **active** physical exercise if he/she has done sports in the last 2 years, this practice has not worsened in the last 6 months by spending at least 150 minutes per week in physical activity.

Supplementary File 3. Dietary composition modifications

(A) Dietary composition modifications (overall)

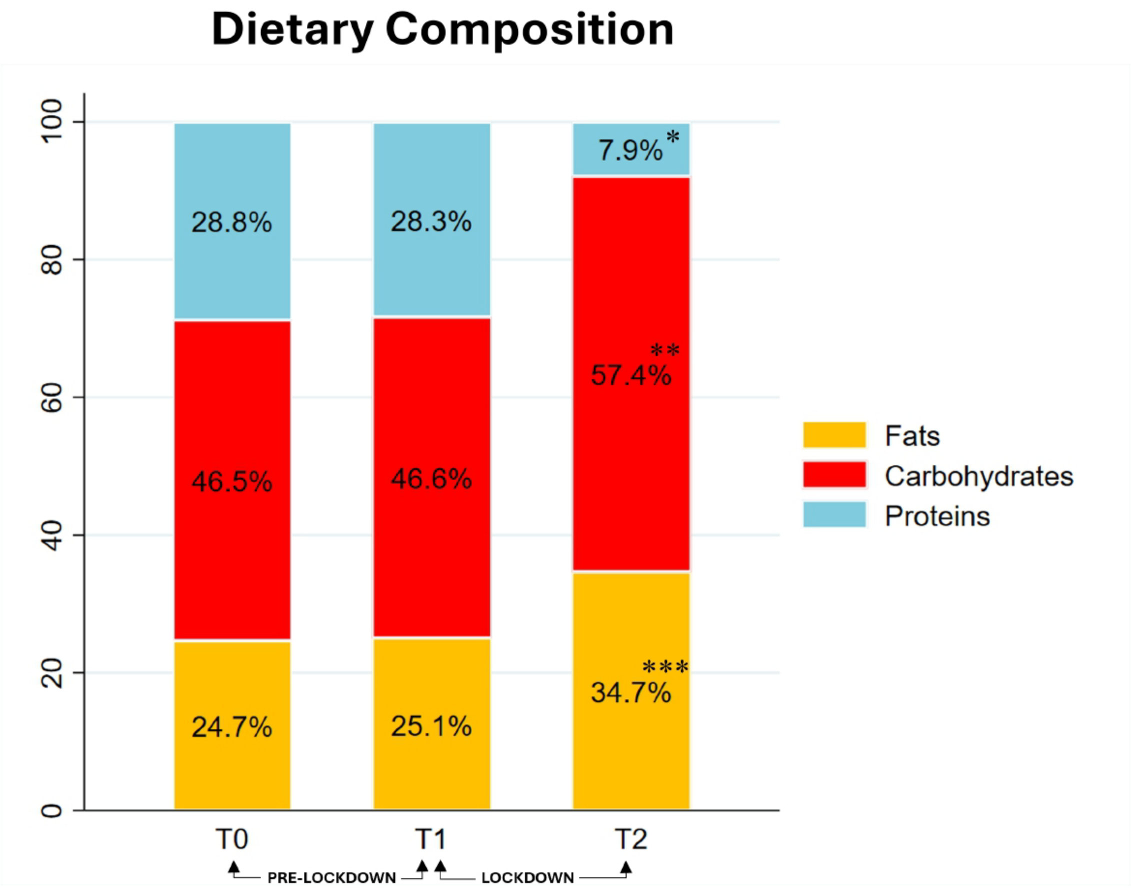

Carbohydrates, **fats, and ***proteins proportions comparison by using ANOVA and Tukey post hoc analysis between baseline and end of the study evaluations, p<0.0001. The intermediate column (T1) indicates the January 2020 time-point assessment.

(B) Dietary composition modifications (fat types)

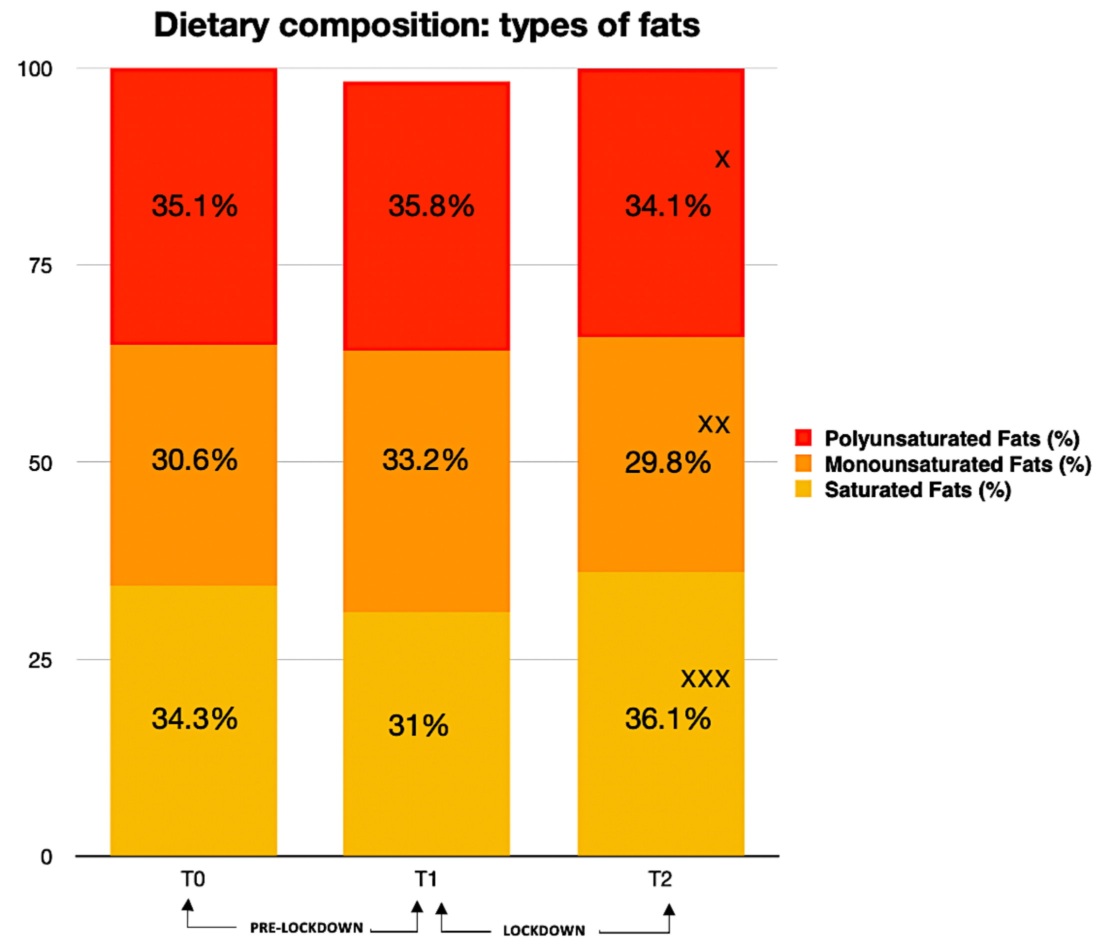

[x] Polyunsaturated fats, [xx] Monounsaturated fats, and [xxx] Polyunsaturated fats' proportions comparison by using ANOVA and Tukey post hoc analysis between baseline and end of the study evaluations. X: not statistically significantly different. The intermediate column (T1) indicates the January 2020 time-point assessment.

(C) Dietary composition modifications (carbohydrate types)

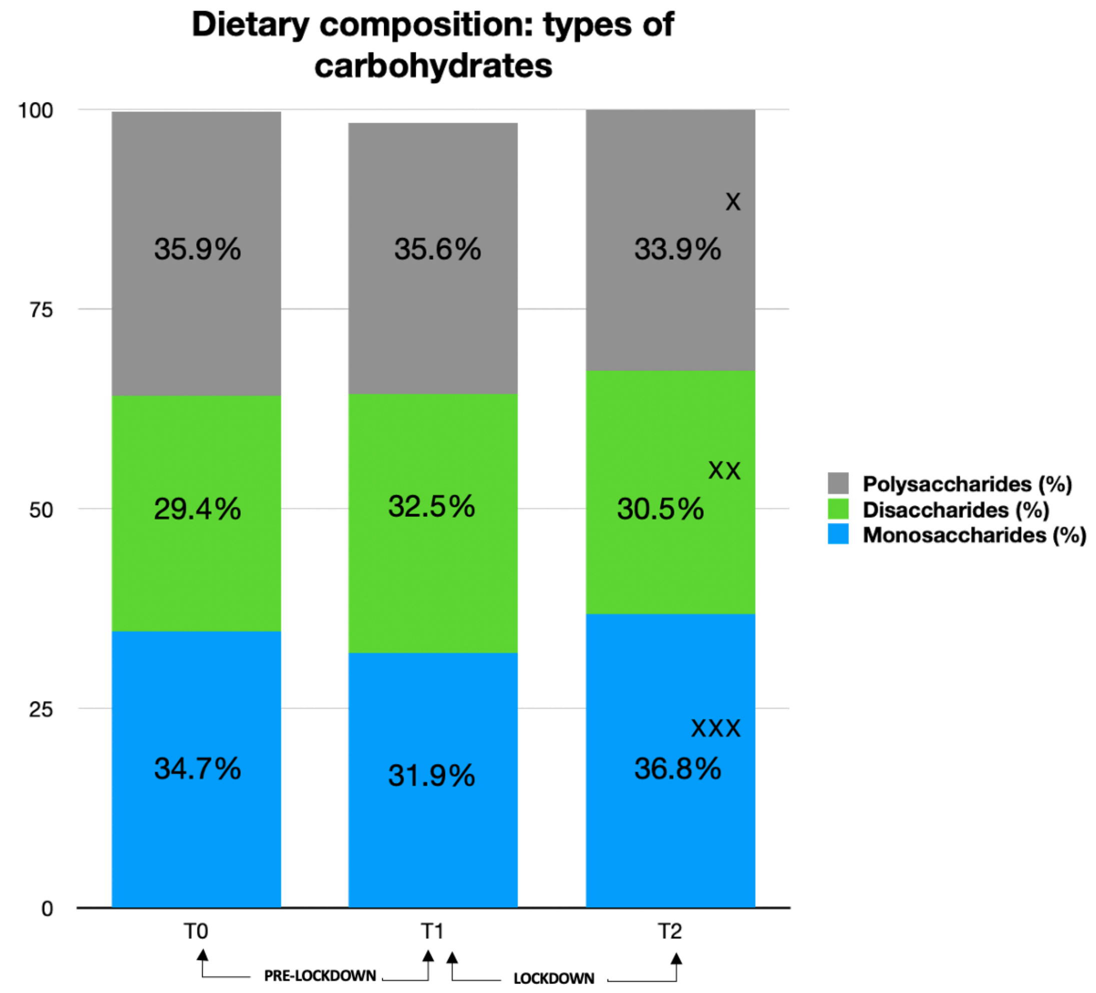

[x] Polysaccharides, [xx] Disaccharides, and [xxx] Monosaccharides' proportions comparison by using ANOVA and Tukey post hoc analysis between baseline and end of the study evaluations. X: not statistically significantly different. The intermediate column (T1) indicates the January 2020 time-point assessment.

Supplementary File 4. The sensitive analysis for main outcomes stratified by age, sex, obesity, diabetes, and Sars-CoV-2.

Assessment of LSM, CAP, and NFS of the study population among the three-time points evaluations by age, sex, BMI, T2D, and SarsCoV2.

LSM (kPa)						
**Variables**	**Baseline (T0: January 2018)**	**Intermediate (T1: January 2020)**	**End of the study (T2: January 2022)**	**Comparison between the three-time points evaluations**		
				**Time-points**	**95% CI**	**p-value**
Age						
< median age	9.44 ± 4.82	9.37 ± 4.71	11.43 ± 5.31	T0 vs T1	-0.03 to 0.17	0.169
				T0 vs T2	-2.56 to -1.78	**<0.001**
				T1 vs T2	-2.63 to -1.84	**<0.001**
≥ median age	10.05 ± 4.38	10.09 ± 4.28	11.86 ± 5.21	T0 vs T1	-0.15 to 0.08	0.560
				T0 vs T2	-2.57 to -1.79	**<0.001**
				T1 vs T2	-2.55 to -1.72	**<0.001**
Sex						
Female	11.17 ± 4.40	11.12 ± 4.34	12.98 ± 5.51	T0 vs T1	-0.07 to 0.18	0.411
				T0 vs T2	-2.44 to -1.61	**<0.001**
				T1 vs T2	-2.52 to -1.63	**<0.001**
Male	8.27 ± 4.31	8.30 ± 4.18	10.27 ± 4.57	T0 vs T1	-0.11 to 0.051	0.443
				T0 vs T2	-2.69 to -1.96	**<0.001**
				T1 vs T2	-2.66 to -1.92	**<0.001**
BMI						
< median BMI	9.42 ± 4.60	9.37 ± 4.60	11.48 ± 5.60	T0 vs T1	-0.04 to 0.13	0.337
				T0 vs T2	-2.52 to -1.55	**<0.001**
				T1 vs T2	-2.55 to -1.58	**<0.001**
≥ median BMI	9.95 ± 4.54	9.98 ± 4.34	11.94 ± 5.31	T0 vs T1	-0.18 to 0.11	0.671
				T0 vs T2	-2.75 to -1.88	**<0.001**
				T1 vs T2	-2.72 to -1.45	**<0.001**
T2D						
No	9.71 ± 4.65	9.78 ± 4.54	11.86 ± 5.18	T0 vs T1	-0.15 to 0.01	0.09
				T0 vs T2	-2.77 to -2.12	**<0.001**
				T1 vs T2	-2.68 to -2.06	**<0.001**
Yes	9.88 ± 4.52	9.74 ± 4.41	11.35 ± 5.36	T0 vs T1	-0.01 to 0.28	0.07
				T0 vs T2	-2.24 to -1.27	**<0.001**
				T1 vs T2	-2.44 to -1.34	**<0.001**
SarsCoV2						
No	9.87 ± 4.58	9.86 ± 4.49	11.77 ± 5.28	T0 vs T1	-0.07 to 0.09	0.822
				T0 vs T2	-2.56 to -1.87	**<0.001**
				T1 vs T2	-2.57 to -1.87	**<0.001**
Yes	6.59 ± 4.63	9.56 ± 4.48	11.47 ± 5.21	T0 vs T1	-0.15 to 0.18	0.833
				T0 vs T2	-2.56 to -1.61	**<0.001**
				T1 vs T2	-2.62 to -1.58	**<0.001**

CAP (db/m)						
**Variables**	**Baseline (T0: January 2018)**	**Intermediate (T1: January 2020)**	**End of the study (T2: January 2022)**	**Comparison between the three-time points evaluations**		
				**Time-points**	**95% CI**	**p-value**
Age						
< median age	290.9 ± 32.88	290.31 ± 34.02	339.39 ± 38.96	T0 vs T1	-1.89 to 3.06	0.638
				T0 vs T2	-55.49 to -41.76	**<0.001**
				T1 vs T2	--55.97 to -42.5	**<0.001**
≥ median age	290.80 ± 32.82	291.27 ± 33.44	329.93 ± 50.53	T0 vs T1	-2.31 to 1.37	0.615
				T0 vs T2	-46.3 to -28.84	**<0.001**
				T1 vs T2	-45.9 to -28.69	**<0.001**
Sex						
Female	288.63 ± 33.49	287.19 ± 32.84	329.67 ± 45.91	T0 vs T1	-0.51 to 3.38	0.145
				T0 vs T2	-47.53 to -31.14	**<0.001**
				T1 vs T2	-48.5 to -32.7	**<0.001**
Male	293.23 ± 32.03	294.77 ± 34.17	339.11 ± 45.29	T0 vs T1	-3.83 to 0.756	0.186
				T0 vs T2	-54.1 to -38.16	**<0.001**
				T1 vs T2	-53.15 to -39.96	**<0.001**
BMI						
< median BMI	290.02 ± 37.29	289.23 ± 36.84	322.98 ± 50.05	T0 vs T1	-1.58 to 3.17	0.509
				T0 vs T2	-49.9 to -25.64	**<0.001**
				T1 vs T2	-49.87 to -29.37	**<0.001**
≥ median BMI	290.67 ± 27.51	291.31 ± 30.65	344.71 ± 35.01	T0 vs T1	-3.25 to 1.98	0.630
				T0 vs T2	-60.24 to -43.4	**<0.001**
				T1 vs T2	-61.02 to -44.46	**<0.001**
T2D						
No	290.05 ± 33.48	288.93 ± 33.89	333.644 ± 43.59	T0 vs T1	-0.66 to 2.91	0.218
				T0 vs T2	-50.32 to -35.5	**<0.001**
				T1 vs T2	-51.36 to -36.91	**<0.001**
Yes	292.05 ± 31.89	293.75 ± 33.19	335.21 ± 49.08	T0 vs T1	-4.33 to 0.92	0.201
				T0 vs T2	-51.31 to -33.12	**<0.001**
				T1 vs T2	-49.78 to -31.59	**<0.001**
SarsCoV2						
No	289.59 ± 33.89	289.76 ± 34.19	334.43 ± 46.71	T0 vs T1	-1.87 to 1.53	0.845
				T0 vs T2	-50.81 to -36.84	**<0.001**
				T1 vs T2	-50.52 to -36.86	**<0.001**
Yes	293.37 ± 30.57	293.02 ± 32.58	333.94 ± 44.12	T0 vs T1	-2.65 to 3.36	0.814
				T0 vs T2	-50.38 to -30.29	**<0.001**
				T1 vs T2	-51.05 to -30.91	**<0.001**

**NFS**						
**Variables**	**Baseline (T0: January 2018)**	**Intermediate (T1: January 2020)**	**End of the study (T2: January 2022)**	**Comparison between the three-time points evaluations**		
				**Time-points**	**95% CI**	**p-value**
Age						
< median age	-1.19 ± 0.84	-1.09 ± 0.89	-0.48 ± 0.98	T0 vs T1	-0.26 to 0.06	0.214
				T0 vs T2	-0.88 to -0.56	**<0.001**
				T1 vs T2	-0.81 to -0.42	**<0.001**
≥ median age	-0.35 ± 0.85	-0.25 ± 0.91	0.34 ± 1.11	T0 vs T1	-0.18 to 003	**0.04**
				T0 vs T2	-0.87 to -0.56	**<0.001**
				T1 vs T2	-0.77 to -0.45	**<0.001**
Sex						
Female	-0.61 ± 0.95	-0.45 ± 0.99	0.11 ± 1.10	T0 vs T1	-0.29 to -0.001	**0.046**
				T0 vs T2	-0.91 to -0.58	**<0.001**
				T1 vs T2	-0.77 to -0.39	**<0.001**
Male	-0.86 ± 0.92	-0.82 ± 0.96	-0.18 ± 1.15	T0 vs T1	-0.13 to 0.05	0.358
				T0 vs T2	-0.84 to -0.54	**<0.001**
				T1 vs T2	-0.81 to -0.49	**<0.001**
BMI						
< median BMI	-0.78 ± 0.94	-0.79 ± 0.99	-0.37 ± 0.98	T0 vs T1	-0.07 to 0.11	0.666
				T0 vs T2	-0.59 to -0.25	**<0.001**
				T1 vs T2	-0.71 to -0.32	**<0.001**
≥ median BMI	-0.56 ± 0.98	-0.34 ± 0.99	0.26 ± 1.21	T0 vs T1	-0.41 to -0.03	**0.02**
				T0 vs T2	-0.96 to -0.54	**<0.001**
				T1 vs T2	-0.84 to -0.48	**<0.001**
T2D						
No	-1.16 ± 0.79	-1.05 ± 0.86	-0.55 ± 0.91	T0 vs T1	-0.24 to 0.02	0.09
				T0 vs T2	-0.77 to -0.48	**<0.001**
				T1 vs T2	-0.67 to -0.33	**<0.001**
Yes	-0.07 ± 0.76	0.01 ± 0.82	0.76 ± 0.97	T0 vs T1	-0.17 to 0.03	0.161
				T0 vs T2	-1.04 to -0.68	**<0.001**
				T1 vs T2	-0.96 to -0.61	**<0.001**
SarsCoV2						
No	-0.73 ± 0.98	-0.64 ± 0.95	-0.09 ± 1.07	T0 vs T1	-0.16 to 0.014	0.09
				T0 vs T2	-0.81 to -0.54	**<0.001**
				T1 vs T2	-0.71 to -0.44	**<0.001**
Yes	-0.71 ± 0.95	-0.61 ± 1.07	0.07 ± 1.24	T0 vs T1	-0.34 to 0.11	0.296
				T0 vs T2	-1.01 to -0.59	**<0.001**
				T1 vs T2	-0.69 to -0.43	**<0.001**

Multi-adjusted hazard ratios (HR) with 95% CI of HCC occurrence.

Lockdown participants (Ref: Pre-lockdown participants)	*n*	*HR (95% CI)*
Age		
< median age	84	5.83 (1.28 – 26.53)
≥ median age	103	2.40 (1.08– 5.33)
Sex		
Female	97	2.59 (0.98 – 6.85)
Male	90	2.34 (0.58 – 9.48)
BMI		
< median BMI	93	2.74 (0.84 – 8.92)
≥ median BMI	94	2.40 (1.08 – 5.31)
Type 2 diabetes		
No	113	1.98 (0.63 – 5.97)
Yes	74	3.23(1.02 – 10.18)
SarsCoV2		
No	125	2.15 (0.85 – 5.41)
Yes	62	3.15 (0.63 – 15.80)
LSM		
< median LSM	94	N/A
≥ median LSM	93	2.39 (1.08 – 5.31)

Model adjusted for age, sex, BMI, type 2 diabetes, SarsCov2, LSM.

Multi-adjusted hazard ratios (HR) with 95% CI of HCC occurrence Milan-out criteria.

Lockdown participants (Ref: Pre-lockdown participants)	*n*	*HR (95% CI)*
Age		
< median age	84	6.50 (0.77 – 54.58)
≥ median age	103	5.91 (1.29– 27.11)
Sex		
Female	97	4.41 (0.91 – 21.38)
Male	90	N/A
BMI		
< median BMI	93	5.42 (0.57 – 51.26)
≥ median BMI	94	5.91 (1.29 – 27.11)
Type 2 diabetes		
No	113	5.14 (0.58 – 46.50)
Yes	74	7.70 (0.92– 64.31)
SarsCoV2		
No	125	4.87 (1.02– 23.09)
Yes	62	N/A
LSM		
< median LSM	94	N/A
≥ median LSM	93	5.91 (1.29 – 27.11)

Model adjusted for age, sex, BMI, type 2 diabetes, SarsCov2, LSM.

Multinomial logistic regression analysis showing the variables significantly associated with HCC overall at diagnosis occurrence during the lockdown.

	FFM (Kg)	FFM (%)	SMMI	BCM (Kg)	BCM (%)	FM (Kg)	FM (%)	LSM
Age								
< median age	**0.78 (0.65 - 0.94)**	**0.59 (0.43 - 0.82)**	**0.48 (0.27 - 0.87)**	**0.47 (0.28 - 0.79)**	**0.66 (0.50 - 0.86)**	**1.68 (1.21 - 2.33)**	**1.69 (1.23 - 2.32)**	**0.84 (0.71 - 0.99)**
≥ median age	**0.63 (0.40 - 0.98)**	0.52 (0.26 - 1.04)	0.33 (0.10 - 1.08)	0.18 (0.03 - 1.15)	**0.52 (0.28 - 0.99)**	1.33 (0.98 - 1.79)	1.91 (0.96 - 3.81)	0.77 (0.55 - 1.08)
Sex								
Female	**0.76 (0.64 - 0.91)**	**0.78 (0.65 - 0.92)**	0.60 (0.35 - 1.03)	**0.54 (0.36 - 0.80)**	**0.73 (0.57 - 0.95)**	**1.16 (1.02 - 1.33)**	**1.29 (1.08 – 1.52)**	0.89 (0.77 - 1.02)
Male	0.81 (0.64 - 1.02)	0.84 (0.69 - 1.09)	**0.38 (0.18 - 0.84)**	**0.27 (0.079 - 0.92)**	**0.47 (0.27 - 0.82)**	2.11 (0.79 – 11.39)	2.24 (0.87 – 12.09)	0.67 (0.42 - 1.06)
BMI								
< median BMI	**0.74 (0.58 - 0.93)**	**0.64 (0.48 - 0.85)**	0.53 (0.27 - 1.05)	**0.35 (0.18 - 0.70)**	0.67 (0.46 - 0.97)	**1.56 (1.18 - 2.06)**	**1.57 (1.18 - 2.09)**	0.86 (0.74 - 1.00)
≥ median BMI	**0.80 (0.66 - 0.97)**	**0.55 (0.33 - 0.90)**	**0.56 (0.32 - 0.98)**	**0.56 (0.35 - 0.87)**	**0.79 (0.49 - 0.98)**	**1.80 (1.03 - 3.14)**	**1.83 (1.11 - 3.01)**	0.88 (0.70 - 1.09)
Type 2 diabetes								
No	**0.81 (0.68 - 0.97)**	**0.56 (0.37 - 0.85)**	**0.58 (0.34 - 0.99)**	**0.46 (0.28 - 0.76)**	**0.59 (0.41 - 0.84)**	**1.76 (1.18 - 2.64)**	**1.79 (1.17 - 2.73)**	0.85 (0.70 - 1.04)
Yes	**0.59 (0.37 - 0.94)**	**0.68 (0.50 - 0.91)**	**0.40 (0.18 - 0.92)**	**0.37 (0.16 - 0.85)**	**0.65 (0.44 - 0.97)**	**1.29 (1.05 - 1.60)**	**1.48 (1.10 - 1.99)**	**0.78 (0.61 - 0.99)**
SarsCoV2								
No	**0.77 (0.65 - 0.91)**	**0.61 (0.45 - 0.83)**	**0.58 (0.36 - 0.95)**	**0.50 (0.33 - 0.75)**	**0.68 (0.52 - 0.89)**	**1.34 (1.12 - 1.61)**	**1.63 (1.21 - 2.21)**	**0.84 (0.73 - 0.97)**
Yes	0.83 (0.67 - 1.03)	**0.69 (0.50 - 0.96)**	0.15 (0.023 - 1.02)	**0.34 (0.13 - 0.90)**	**0.51 (0.29 - 0.90)**	1.50 (0.99 - 2.29)	**1.45 (1.04 - 2.01)**	0.88 (0.70 - 1.11)
LSM								
< median LSM	**0.76 (0.63 - 0.92)**	**0.65 (0.51 - 0.82)**	**0.43 (0.24 - 0.78)**	**0.30 (0.14 - 0.64)**	**0.59 (0.42 - 0.82)**	**1.53 (1.20 - 1.96)**	**1.55 (1.22 - 1.98)**	0.69 (0.46 - 1.04)
≥ median LSM	0.84 (0.69 - 1.02)	**0.74 (0.55 - 0.99)**	0.52 (0.21 - 1.31)	**0.65 (0.44 - 0.97)**	0.73 (0.50 - 1.05)	1.17 (0.96 - 1.42)	**1.36 (1.01 - 1.82)**	0.85 (0.64 - 1.14)

Model adjusted for age, sex, BMI, type 2 diabetes, SarsCov2, LSM.

Multinomial logistic regression analysis showing the variables significantly associated with HCC Milan-out criteria overall at diagnosis occurrence during the lockdown.

	FFM (Kg)	FFM (%)	SMMI	BCM (Kg)	BCM (%)	FM (Kg)	FM (%)	LSM
Age								
< median age	0.82 (0.66 - 1.01)	**0.35 (0.14 - 0.91)**	0.57 (0.27 - 1.17)	**0.40 (0.16 - 0.97)**	**0.63 (0.41 - 0.95)**	**5.44 (1.11 - 26.7)**	**2.85 (1.10 - 7.38)**	0.86 (0.70 - 1.06)
≥ median age	N/A	N/A	N/A	0.37 (0.093 - 1.46)	0.78 (0.38 - 1.61)	2.25 (0.40 - 12.7)	N/A	0.37 (0.11 - 1.24)
Sex								
Female	**0.76 (0.59 - 0.98)**	**0.70 (0.51 - 0.95)**	0.52 (0.23 - 1.16)	**0.57 (0.33 - 0.98)**	0.80 (0.57 - 1.13)	1.27 (1.00 - 1.60)	**1.43 (1.05 - 1.96)**	0.83 (0.67 - 1.03)
Male	0.75 (0.51 - 1.09)	N/A	0.36 (0.089 - 1.42)	N/A	0.23 (0.032 - 1.72)	N/A	N/A	0.56 (0.24 - 1.33)
BMI								
< median BMI	0.42 (0.13 - 1.40)	N/A	0.18 (0.0044 - 6.96)	0.42 (0.14 - 1.32)	0.67 (0.34 - 1.29)	N/A	N/A	0.58 (0.30 - 1.13)
≥ median BMI	0.67 (0.43 - 1.04)	N/A	0.34 (0.089 - 1.30)	N/A	N/A	N/A	N/A	0.89 (0.66 - 1.19)
Type 2 diabetes								
No	0.85 (0.68 - 1.05)	**0.53 (0.29 - 0.97)**	0.66 (0.35 - 1.24)	**0.50 (0.28 - 0.90)**	**0.47 (0.23 - 0.95)**	**1.99 (1.07 - 3.72)**	**1.90 (1.04 - 3.49)**	0.90 (0.68 - 1.17)
Yes	0.29 (0.058 - 1.49)	N/A	0.28 (0.061 - 1.27)	0.57 (0.28 - 1.14)	0.94 (0.62 - 1.42)	4.22 (0.61 - 29.0)	N/A	0.65 (0.41 - 1.02)
SarsCoV2								
No	**0.82 (0.69 - 0.98)**	**0.63 (0.46 - 0.87)**	0.71 (0.41 - 1.23)	**0.60 (0.40 - 0.89)**	**0.75 (0.57 - 0.99)**	**1.41 (1.10 - 1.80)**	**1.59 (1.15 - 2.19)**	0.84 (0.70 - 1.01)
Yes	0.80 (0.53 - 1.21)	0.63 (0.29 - 1.37)	N/A	0.13 (0.0033 - 5.32)	0.47 (0.17 - 1.30)	1.77 (0.72 - 4.36)	1.59 (0.73 - 3.48)	0.71 (0.37 - 1.36)
LSM								
< median LSM	**0.78 (0.62 - 0.99)**	**0.67 (0.51 - 0.89)**	**0.46 (0.23 - 0.95)**	**0.45 (0.25 - 0.83)**	**0.68 (0.48 - 0.97)**	**1.41 (1.11 - 1.79)**	**1.48 (1.12 - 1.96)**	0.78 (0.48 - 1.27)
≥ median LSM	0.87 (0.67 - 1.14)	0.70 (0.43 - 1.13)	0.76 (0.19 - 3.01)	0.78 (0.48 - 1.28)	0.82 (0.49 - 1.35)	1.32 (0.87 - 1.99)	1.43 (0.88 - 2.33)	0.63 (0.38 - 1.06)

Model adjusted for age, sex, BMI, type 2 diabetes, SarsCov2, LSM.

Supplementary Table 1. Assessment of physical activity, nutritional and dietary habits among the three-time points evaluations

**Variables** (mean ± SD)	**Baseline (T0: January 2018)**	**Intermediate (T1: January 2020)**	**End of the study (T2: January 2022)**	**Comparison between the three-time points evaluations**		
				**Time-points**	**95% CI**	**p-value**
Physical activity (hours/week)	6.1 ± 1.3	6.1 ± 1.2	4.3 ± 1.4	T0 vs T1	-0.2026 to 0.2206	0.993
				T0 vs T2	1.529 to 2.201	**<0.0001**
				T1 vs T2	1.521 to 2.189	**<0.0001**
Alcohol intake* (“Drink”/day)	1.4 ± 0.3	1.7 ± 0.2	1.5 ± 0.4	T0 vs T1	-51.90 to 61.39	0.989
				T0 vs T2	-54.20 to 61.47	0.994
				T1 vs T2	-56.30 to 64.81	0.991
Daily intake (Kilocalories / day)	2296 ± 397.1	2292 ± 401.9	3024 ± 545.4	T0 vs T2	-59.20 to 67.49	0.986
				T1 vs T2	-847.3 to -607.6	**<0.0001**
				T1 vs T2	- 845.4 to -617.8	**<0.0001**
Carbohydrates (Kilocalories)	1069 ± 227	1069 ± 235	1716 ± 414.1	T0 vs T1	-36.49 to 36.64	> 0.99
				T0 vs T2	-727.5 to -566.1	**<0.0001**
				T1 vs T2	-727.5 to -566.2	**<0.0001**
Lipids (Kilocalories)	566.6 ± 126.9	574.4 ± 126.7	1055 ± 261.4	T0 vs T1	-28.72 to 13.17	0.655
				T0 vs T2	-538.5 to -438.1	**<0.0001**
				T1 vs T2	-528.9 to -432.2	**<0.0001**
Proteins (Kilocalories)	661 ± 185.1	649.2 ± 198.9	234.3 ± 166.9	T0 vs T1	-18.18 to 41.86	0.62
				T0 vs T2	387 to 466.5	**<0.0001**
				T1 vs T2	375.4 to 454.5	**<0.0001**

Assessed by using AUDIT-C test (one drink corresponds to about 12 g of pure ethanol); SD: standard deviation. The Kruskal-Wallis test or ANOVA test with post-hoc Tukey analysis, in the case of non-normal or normal distribution respectively, were performed to compare the continuous variables among three observation times. Statistically significant differences (p<0.05) among the three periods are reported in bold.*

Supplementary Table 2. Assessment of body composition parameters among the three-time points evaluations

Variables (mean ± SD)	Baseline (T0: January 2018)	Intermediate (T1: January 2020)	End of the study (T2: January 2022)	Comparison between the three-time points evaluations		
				Time-points	95% CI	p-value
FFM (Kg)	63.4 ± 7.9	63.3 ± 8.2	63.7 ± 11.1	T0 vs T1	-0.388 to 0.624	0.847
				T0 vs T2	-1.973 to 1.263	0.862
				T1 vs T2	-2.082 to 1.138	0.767
FFM (%)	79.7 ± 4	79.7 ± 3.9	74 ± 6.3	T0 vs T1	-0.348 to 0.313	0.991
				T0 vs T2	4.628 to 6.586	**<0.0001**
				T1 vs T2	4.677 to 6.572	**<0.0001**
SMMI (Kg/m^2)	10.4 ± 1.1	10.3 ± 1.1	10.4 ± 1.6	T0 vs T1	-0.067 to 0.079	0.979
				T0 vs T2	-0.286 to 0.188	0.877
				T1 vs T2	-0.291 to 0.181	0.846
FM (Kg)	16.2 ± 3.9	16.2 ± 3.9	22.5 ± 7.6	T0 vs T1	-0.286 to 0.358	0.962
				T0 vs T2	-7.534 to -5.106	**<0.0001**
				T1 vs T2	-7.506 to -5.205	**<0.0001**
FM (%)	20.3 ± 4.01	20.3 ± 3.9	26 ± 6.3	T0 vs T1	-0.314 to 0.346	0.992
				T0 vs T2	-6.593 to -4.635	**<0.0001**
				T1 vs T2	-6.557 to -4.683	**<0.0001**
ECM (Kg)	31.3 ± 5.8	31.3 ± 5.8	31.1 ± 7.2	T0 vs T1	-0.306 to 0.461	0.882
				T0 vs T2	-2.816 to -0.721	**0.0003**
				T1 vs T2	-2.875 to - 0.817	**0.0001**
ECM (%)	39.26 ± 4.8	39.3 ± 4.7	35.9 ± 6.2	T0 vs T1	-0.347 to 0.379	0.994
				T0 vs T2	-3.148 to -1.881	**<0.0001**
				T1 vs T2	-3.035 to -2.026	**<0.0001**
BCM (Kg)	25.5 ± 3.4	25.5 ± 3.6	24.1 ± 4.9	T0 vs T1	-0.253 to 0.333	0.944
				T0 vs T2	0.644 to 2.184	**<0.0001**
				T1 vs T2	0.661 to 2.088	**<0.0001**
BCM (%)	40.4 ± 3.5	40.3 ± 3.5	37.8 ± 4	T0 vs T1	-0.379 to 0.347	0.994
				T0 vs T2	1.881 to 3.148	**<0.0001**
				T1 vs T2	2.026 to 3.035	**<0.0001**
TBW (%)	46.3 ± 5.9	46.1 ± 5.8	46.7 ± 7.3	T0 vs T1	-0.667 to 1.056	0.855
				T0 vs T2	-1.742 to 0.986	0.79
				T1 vs T2	-1.971 to 0.828	0.599

BCM: body cell mass; ECM: extracellular mass; FM: fat mass; FFM: free fat mass; SMM: skeletal muscle mass; SMMI: skeletal muscle mass index; SD: standard deviation; TBW: total body water. Statistically significant differences between the three periods are reported in bold. The Kruskal-Wallis test or ANOVA test with post-hoc Tukey analysis, in the case of non-normal or normal distribution respectively, were performed to compare the continuous variables among three times of observation. Statistically significant differences (p<0.05) among the three periods are reported in bold

Supplementary Table 3. (A) Test of proportional hazards assumption for HCC occurrence.

	**Model 1***			
	rho	*chi2*	*df*	*Prob>chi2*
Lockdown participants (Ref: Pre-lockdown participants)	0.03376	0.03	1	0.8583
Global test	.	0.03	1	0.8583

	**Model 2ⴕ**			
	rho	*chi2*	*df*	*Prob>chi2*
Lockdown participants (Ref: Pre-lockdown participants)	0.03270	0.03	1	0.8638
Age	-0.09254	0.19	1	0.6597
Male (Ref: female)	0.02120	0.02	1	0.9017
BMI	0.09725	0.35	1	0.5549
Type 2 diabetes (Ref: not type 2 diabetes)	-0.31859	2.92	1	0.0876
SarsCov2 (Ref: not SarsCov2)	0.12449	0.46	1	0.4984
LSM	0.11117	0.50	1	0.4806
Global test	.	4.48	7	0.7233

*Model 1: not adjusted.

ⴕModel 2: adjusted for age, sex, BMI, type 2 diabetes, SarsCov2, LSM.

(B) Test of proportional hazards assumption for HCC occurrence Milan-out criteria

	**Model 1***			
	rho	*chi2*	*df*	*Prob>chi2*
Lockdown participants (Ref: Pre-lockdown participants)	-0.51209	3.13	1	0.0771
Global test	.	3.13	1	0.0771

	**Model 2⸸**			
	rho	*chi2*	*df*	*Prob>chi2*
Lockdown participants (Ref: Pre-lockdown participants)	-0.51444	3.04	1	0.0814
Age	-0.11495	0.13	1	0.7218
Male (Ref: female)	-0.02628	0.01	1	0.9301
BMI	0.10895	0.13	1	0.7137
Type 2 diabetes (Ref: not type 2 diabetes)	-0.33285	1.30	1	0.2535
SarsCov2 (Ref: not SarsCov2)	-0.10268	0.13	1	0.7220
LSM	0.51544	2.12	1	0.1449
Global test		7.27	7	0.4015

*Model 1: not adjusted.

⸸Model 2: adjusted for age, sex, BMI, type 2 diabetes, SarsCov2, LSM.

Supplementary Table 4. Multinomial logistic regression analysis showing the variables significantly associated with HCC overall and HCC staged Milan-out criteria at diagnosis occurrence during the lockdown. The odds ratios (OR) of the study variables on the just mentioned events were calculated considering the confounding variables (age, sex, BMI, T2DM, SARS-CoV-2 infection, and LSM).

**Outcome: HCC overall occurrence during the lockdown**			
**Variable**	**Odds ratio**	**Confidence Interval (95%)**	**p-value**
FFM (Kg)	0.809	0.72-0.909	0.0003
FFM (%)	0.703	0.597-0.828	<0.0001
SMMI	0.568	0.387-0.834	0.004
BCM (Kg)	0.536	0.401-0.718	<0.0001
BCM (%)	0.664	0.531-0.83	0.0003
FM (Kg)	1.33	1.159-1.527	<0.0001
FM (%)	1.422	1.207-1.675	<0.0001
LSM (kPa)	0.851	0.752-0.963	0.01
**Outcome: HCC staged Milan-out criteria at diagnosis during the lockdown**			
**Variable**	**Odds ratio**	**Confidence Interval (95%)**	**p-value**
FFM (Kg)	0.812	0.694-0.951	0.01
FFM (%)	0.687	0.542-0.87	0.002
SMMI	0.596	0.362-0.979	0.04
BCM (Kg)	0.583	0.408-0.833	0.003
BCM (%)	0.717	0.549-0.936	0.01
FM (Kg)	1.363	1.121-1.656	0.002
FM (%)	1.456	1.15-1.845	0.002
LSM (kPa)	0.822	0.688-0.982	0.03

BCM: Body cellular mass; FFM: Free fat mass; FM: Fat mass; LSM: Liver stiffness measurement; kPa: Kilopascal; Kg: kilograms; SMM: Skeletal muscle mass; SMMI: Skeletal muscle mass index.
